# Postprandial Circulating miRNAs in Response to a Dietary Fat Challenge

**DOI:** 10.3390/nu11061326

**Published:** 2019-06-13

**Authors:** Diana C. Mantilla-Escalante, María-Carmen López de las Hazas, Judit Gil-Zamorano, Lorena del Pozo-Acebo, M. Carmen Crespo, Roberto Martín-Hernández, Andrea del Saz, Joao Tomé-Carneiro, Fernando Cardona, Isabel Cornejo-Pareja, Almudena García-Ruiz, Olivier Briand, Miguel A. Lasunción, Francesco Visioli, Alberto Dávalos

**Affiliations:** 1 Laboratory of Epigenetics of Lipid Metabolism, Madrid Institute for Advanced Studies (IMDEA)-Food, CEI UAM + CSIC, 28049 Madrid, Spain; diana.mantilla@imdea.org (D.C.M.-E.); mcarmen.lopez@imdea.org (M.-C.L.d.l.H.); judit.gil@imdea.org (J.G.-Z.); lorena.delpozo@imdea.org (L.d.P.-A.); Andrea.Saz@alu.uclm.es (A.d.S.); almudena.garcia@imdea.org (A.G.-R.); 2 Laboratory of Functional Foods, Madrid Institute for Advanced Studies (IMDEA)-Food, CEI UAM + CSIC, 28049 Madrid, Spain; carmen.crespo@imdea.org (M.C.C.); joao.estevao@imdea.org (J.T.-C.); francesco.visioli@imdea.org (F.V.); 3 GENYAL Platform on Nutrition and Health, Madrid Institute for Advanced Studies (IMDEA)-Food, CEI UAM + CSIC, 28049 Madrid, Spain; roberto.martin@imdea.org; 4 Unidad de Gestión Clínica de Endocrinología y Nutrición del Hospital Virgen de la Victoria, Instituto de Investigación Biomédica de Málaga (IBIMA), Universidad de Málaga, 29010 Málaga, Spain; fernandocardonadiaz@gmail.com (F.C.); isabelmaria_cornejo@hotmail.com (I.C.-P.); 5 CIBER Fisiopatología de la Obesidad y Nutrición (CIBEROBN), Instituto de Salud Carlos III, 28029 Madrid, Spain; miguel.a.lasuncion@hrc.es; 6 Univ. Lille, Inserm, CHU Lille, Institut Pasteur de Lille, U1011- EGID, F-59000 Lille, France; olivier.briand@univ-lille.fr; 7 Servicio de Bioquímica Investigación, Hospital Universitario Ramón y Cajal, IRYCIS, 28034 Madrid, Spain; 8 Department of Molecular Medicine, University of Padova, 35121 Padova, Italy

**Keywords:** circulating miRNA, postprandial, fat challenge, diet, exosomes

## Abstract

Postprandial lipemia has many physiopathological effects, some of which increase the risk of cardiovascular disease. MicroRNAs (miRNAs) can be found in almost all biological fluids, but their postprandial kinetics are poorly described. We aimed to profile circulating miRNAs in response to a fat challenge. In total, 641 circulating miRNAs were assessed by real-time PCR in plasmas from mice two hours after lipid gavage. Mice with intestine-specific loss of Dicer were screened to identify potential miRNAs released by the intestine. A total of 68 miRNAs were selected for further validation. Ten circulating miRNAs were finally validated as responsive to postprandial lipemia, including miR-206-3p, miR-543-3p, miR-466c-5p, miR-27b-5p, miR-409-3p, miR-340-3p, miR-1941-3p, miR-10a-3p, miR-125a-3p, and miR-468-3p. Analysis of their possible tissues of origin/target showed an enrichment of selected miRNAs in liver, intestine, brain, or skeletal muscle. miR-206, miR-27b-5p, and miR-409-3p were validated in healthy humans. Analysis of their predicted target genes revealed their potential involvement in insulin/insulin like growth factor (insulin/IGF), angiogenesis, cholecystokinin B receptor signaling pathway (CCKR), inflammation or Wnt pathways for mice, and in platelet derived growth factor (PDGF) and CCKR signaling pathways for humans. Therefore, the current study shows that certain miRNAs are released in the circulation in response to fatty meals, proposing them as potential novel therapeutic targets of lipid metabolism.

## 1. Introduction

Cardiovascular diseases (CVD) are the leading cause of morbidity and mortality in the world [1]. The residual risk of continuing to suffer from this metabolic disorder, even after using all current therapies, remains high [2]. Although lifestyle changes such as diet and exercise generally help, they are often insufficient to achieve healthy lipid profiles, requiring the use of therapeutic treatments to improve plasma lipid levels [3]. 

The postprandial state is a dynamic period of metabolic traffic, biosynthesis, and oxidative metabolism of absorbed substrates such as glucose, lipids, proteins, and other biomolecules contained in the diet. During this period, the body responds with compensatory and adaptive mechanisms and manages short-term disturbances to restore homeostasis. Developed countries often use high-energy diets and exhibit limited energy expenditure, resulting in prolonged metabolic, oxidative, and immune imbalance [4], associated with CVD development [5]. 

Postprandial lipemia is characterized by the enhancement of triglyceride (TG)-rich lipoprotein production. The concentration and duration of the postprandial TG response is closely associated with a higher risk of atherosclerosis [6]. Some dietary factors, such as food consumption, composition of macro and micronutrients, and lipidic factors (lipid quantity, fatty acid composition, and TG structure), modulate postprandial lipemia [7]. 

The mechanisms involved in postprandial lipemia remain poorly understood [8]; therefore, it is necessary to understand and discover new molecular mechanisms of lipid metabolism. One example is the identification of epigenetic actors such as microRNAs (miRNAs), which are small noncoding RNAs that negatively regulate gene expression. The role of miRNAs in the posttranscriptional regulation of lipid metabolism highlights their potential for future diagnostic and even therapeutic applications against lipid-related disorders [9]. Diet is a critical parameter to be considered while determining cardiovascular risk [10], and increasing evidence suggests that miRNAs can be therapeutically modulated by diet [11]. Thus, the identification of miRNAs modulated under a postprandial lipemia state may help in finding new therapeutic targets to prevent CVD [12]. In addition to intracellular locations, miRNAs can also be found in almost all biological fluids, including serum [13]. Extracellular miRNAs participate in intercellular communication by regulating gene expression of the recipient cells [14]. Although the biological function of most circulating miRNAs still remains to be determined, they have been suggested to be promising biomarkers of health and disease [15].

Recently, the modulation of circulating miRNAs (c-miRNAs) through dietary compounds [16] and diets [17,18] has been described. Some studies affirm that environmental factors, such as diet and exercise, influence the epigenetic mechanisms that affect the response to postprandial lipemia [19], but epigenetic factors, such as c-miRNAs, are poorly characterized [20]. Previous studies have evaluated the postprandial regulation of tissue miRNAs in different models, including miRNAs involved in insulin pathways in fish liver [21], or in endothelial cells in response to triglyceride-rich lipoproteins isolated from subjects after consumption of a high-fat meal [22]. In addition, in response to a postprandial high-saturated-fat challenge in healthy individuals, changes in a signature of miRNAs in peripheral blood mononuclear cells were reported [23]. However, there is scarce information about the levels of circulating miRNAs in response to postprandial dietary fat challenge. While one study in the rainbow trout fish showed that circulating miRNAs-128 and miR-223 are subjected to postprandial regulation and might reflect cholesterol-based changes in the liver [24], another study in humans reported an increase of miR-92a and miR-223 in ApoB-depleted serum after consumption of a high-fat meal [25]. These two studies evaluated specific miRNAs; however, there is very little information about postprandial c-miRNAs at a larger scale. Therefore, we sought to evaluate the expression of c-miRNAs in response to postprandial lipemia and to provide a signature of postprandial circulating miRNAs. Different miRNAs that regulate systemic lipid metabolism are modulated in relevant tissues of lipidic systemic control, including the liver and intestine [26,27]. The use of specific deletion of Dicer1, the rate-limiting enzyme in the canonical pathway of miRNAs synthesis [28], allows the studying of tissue-derived secreted miRNAs [29]. Considering that fat is absorbed in the small intestine [30], we also evaluated the possible source of postprandial miRNAs that might be associated with this tissue by using mice intestine-specific Dicer1 deletion. 

## 2. Materials and Methods

### 2.1. Animal Models 

**Figure 1:**
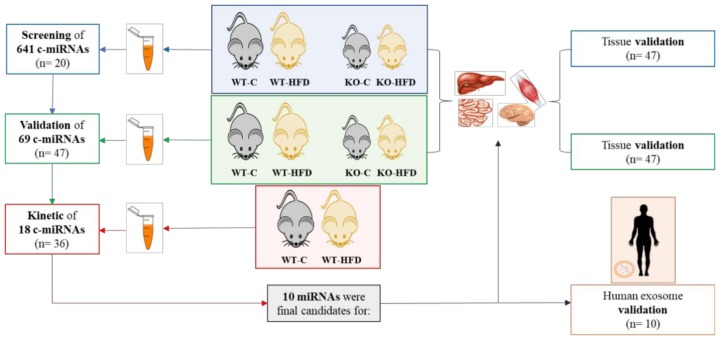
Experimental design of the study. C-miRNAs, circulating microRNAs; WT, wild type; KO, Dicer1-deficient mice; C, control; HFD, oral high fat dietary challenge.

The animal procedures were conducted in accordance with the guidelines of the European Communities Directive 86/609/EEC regulating animal research and approved by the Animal Ethics Committee (Proex 281/15 and Proex 282/15) of Hospital Ramón y Cajal (Madrid, Spain). The experimental design is outlined in Figure 1.

Male C57BL/6 mice were purchased from Charles River (Écully, France). Intestine-specific Dicer1 knockout mice (Dicer1-deficient mice, KO or Dicer1 mutant) were generated by backcrossing Dicer1loxP/loxP (The Jackson Laboratories, Bar Harbor, ME, USA) and Villin-cre mice (The Jackson Laboratories). Dicer1 mutant and their littermates (C57BL/6J control) aged 10–14 weeks old were used in all experiments. Mice were housed in a standard animal facility maintained in regular temperature-controlled rooms (25 ± 2 °C) with controlled lighting (12 h light–dark cycles). Food and water were available ad libitum. 

### 2.2. High-Fat Dietary Challenge

Animals from both genetic backgrounds: C57BL/6 mice (wild type; WT) and Dicer1 knockout mice were divided into two experimental groups according to their treatment: control (C) or oral high-fat dietary challenge (HFD). Both sexes were employed to perform the experiments. The HFD consisted of the administration (oral gavage) of 250 µL olive oil enriched with 40 mg of cholesterol, while the control group received 300 µL of water. Two hours after dietary administration, mice were anesthetized with a mixture of ketamine/xylazine and sacrificed by exsanguination. After that, mice were perfused with Phosphate-Buffered Saline (PBS) to remove all remaining blood. 

### 2.3. Sample Collection

Blood were immediately collected in EDTA and centrifuged at 1500× *g* for 15 min at 4 °C to obtain plasma, which was stored at −80 °C. Small intestine, liver, brain, and skeletal muscle were immediately frozen in liquid nitrogen and stored at −80 °C.

### 2.4. Plasma miRNAs Determination

#### 2.4.1. C-miRNA Screening

Total RNA was isolated from 200 µL of plasma using miRCURY™ RNA Isolation Kit—Biofluids (Exiqon, Denmark) and cDNA synthesized using Universal cDNA synthesis kit II (Exiqon, Denmark) following the manufacturer’s instructions. C-miRNAs were screened using mouse miRNome panels (641 unique mice mature miRNAs) Version 3 (Exiqon, Denmark) by real-time PCR (RT-qPCR) using the ExiLENT SYBR green master mix kit (Exiqon, Denmark) on a 7900HT fast real-time PCR system (Applied Biosystems, Foster City, CA, USA). The relative expression level of each miRNA was determined by normalizing the data with an internal control spike-in (Uni-sp2, Uni-sp4, Uni-sp5, and Uni-sp6). The relative expression was obtained using GenEx Pro analysis software (MultiD Analyses AB, Sweden).

#### 2.4.2. Individual C-miRNA Analysis

Total RNA was isolated from 200 µL of plasma using miRCURY™ RNA Isolation Kit—Biofluids (Exiqon, Denmark), and cDNA was synthesized using Universal cDNA synthesis kit II (Exiqon, Denmark). RT-qPCR was performed using ExiLENT SYBR Green Master Mix (Exiqon, Denmark) and specific miRNAs oligos (Isogen LifeSciences, Utrecht, Nederland) in a 7900HT (Applied Biosystems) thermal cycler. The relative expression level of each miRNA was determined by normalizing the data with the spike-in small RNA cel-miR-39-3p. The expression of c-miRNAs was calculated using GenEx Pro analysis software (MultiD Analyses AB, Sweden). 

#### 2.4.3. Analysis of miRNAs in Tissues

Frozen tissues were disaggregated using TissueRuptor II (Qiagen, Denmark). Total RNA was isolated from 100 mg of tissue using Qiazol Lysis Reagent (Qiagen, Denmark) by the chloroform/phenol method [31]. miRNAs were analyzed as previously described in Section 2.4.2. Reactions were run in duplicate and the relative expression of these selected genes was calculated by the 2^−∆∆Ct^ method using RNU1A, RNU43, and RNU6B as reference genes for normalization.

### 2.5. Human C-miRNA Validation 

#### 2.5.1. Human Study

All participants gave written informed consent and the study was reviewed and approved by the Ethics and Research Committee of Virgen de la Victoria Hospital. Ten healthy subjects, after fasting for 12 h, received an oral fat load based on a preparation of 100 mL containing 50 g of fat consisting of 30% saturated, 49% monounsaturated, and 21% polyunsaturated fatty acids. This preparation contained 1 g of lauric acid, 1 g of myristic acid, 4.8 g of palmitic acid, 1.4 g of stearic acid, 27.7 g of oleic acid, 9.6 g of linoleic acid, 1.4 g of behenic acid, and 0.5 g of lignoceric acid (patent no. P201030776). Only water was permitted during the fat challenge test, and no physical exercise was undertaken. Blood samples were obtained from the antecubital vein and placed in vacutainer tubes (BD Vacutainer™) at baseline and 3 h after the fat load. The samples were immediately frozen at −80 °C until analysis. All participants received nutritional recommendations to follow a similar diet regarding carbohydrate, protein, and lipid percentages on the day before the fat load. 

#### 2.5.2. Exosome Isolation

Exosomes were isolated from 500 μL of plasma using the miRCURY Exosome Serum/Plasma Kit—Biofluids (Qiagen, Denmark) according to the manufacturer’s protocol. Exosome RNA extraction and miRNA analysis were performed as described in Section 2.4.2.

#### 2.5.3. Biochemical Analysis

Total plasma cholesterol and triglycerides were analyzed by standard colorimetric assays (Spinreact, Spain) following the manufacturer’s instructions.

### 2.6. Bioinformatic Analysis

To predict each miRNA’s possible tissue of origin, a hierarchical clustering analysis was performed with the selected subset of miRNAs, using the tissue expression data from the Human miRNA Tissue Atlas (1.0) database [32].

Target genes from the selected differentially expressed miRNAs were obtained from the miRWalk v.2.0 database [33]. Three and ten selected miRNAs, corresponding to human and mouse, respectively, were screened for common target genes. Without any filtering step, 4652 and 638 targets were obtained, respectively, considering interaction sites along 3´untranslated regions (3′UTR), 5´untranslated regions (5′UTR) and coding sequence (CDS) regions. These targets served as input for the functional analysis of affected pathways using the Panther database with default parameters.

In order to build a high-confidence miRNA–mRNA interaction network, and due to the high number of commonly targeted genes, some filtering steps were applied to the previously identified miRNA target genes. For mouse miRNAs, the included target genes interact along the 3′UTR transcript region, with a binding probability equal to 1, and a miRNA–mRNA thermodynamic binding energy lower than −20 kcal/mol. The resulting interaction network includes 152 genes simultaneously targeted by at least 4 miRNAs. Target dot sizes are directly correlated with the number of interactions with the miRNA’s set. For human miRNAs, the interaction network includes interactions on the 3′UTR region with a binding probability equal to 1. The resulting interaction network includes 42 genes simultaneously targeted by the 3 miRNAs. Plots were obtained using the software Cytoscape v.3.6.0. (New York, NY, USA).

### 2.7. Statistical Study 

**Figure 2:**
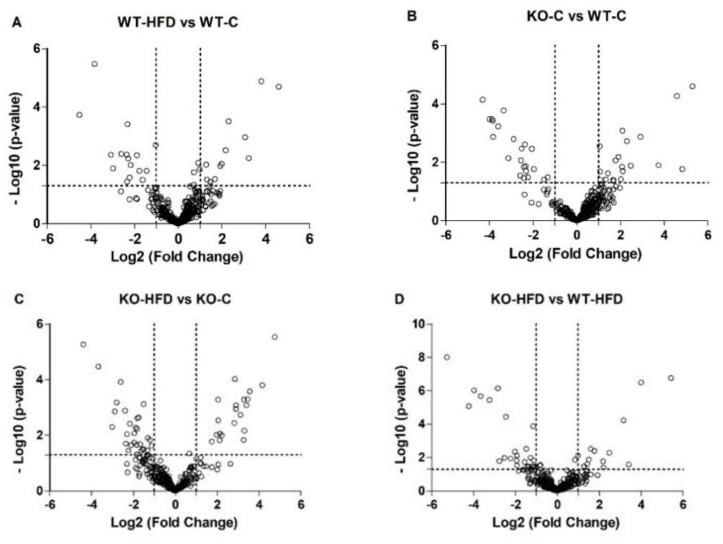
Screening of miRNAs modulated by oral dietary fat challenge (HFD). Volcano plots of modulated miRNAs between: (**A**) WT-C vs. WT-HFD; (**B**) WT-C vs. KO-C; (**C**) KO-C vs. KO-HFD; and (**D**) WT-HFD vs. KO-HFD. miRNA expression analyzed by RT-qPCR (n = 5 mice per group). WT, wild type; KO, Dicer1-deficient mice; C, control; HFD, oral high-fat dietary challenge.

**Figure 3:**
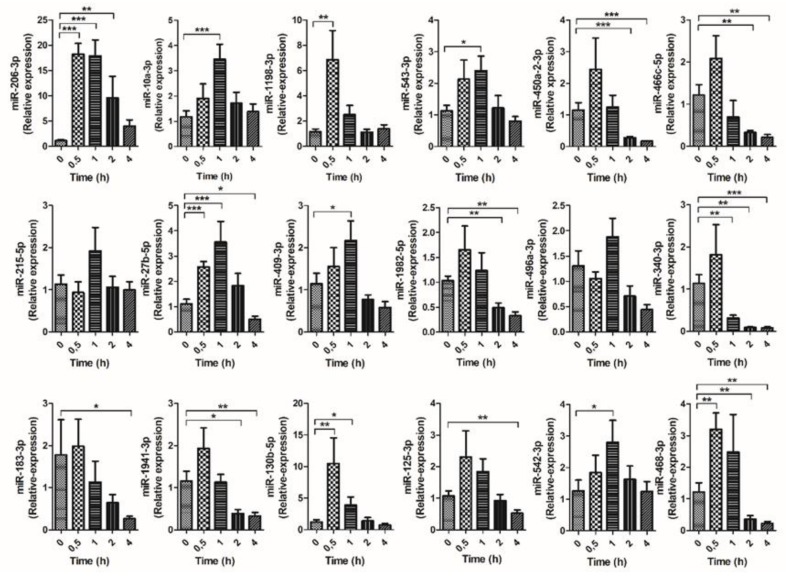
Time-course expression of circulating miRNAs in response to an oral high-fat dietary challenge (HFD). Male C57Bl/6J male mice were administered the HFD and miRNAs were analyzed at different time points (0, 0.5, 1, 2, and 4 h). RT-qPCR analysis of selected miRNAs was significantly different from controls (time 0 h) at * *p* < 0.05; ** *p* < 0.01; *** *p* < 0.001.

**Figure 4:**
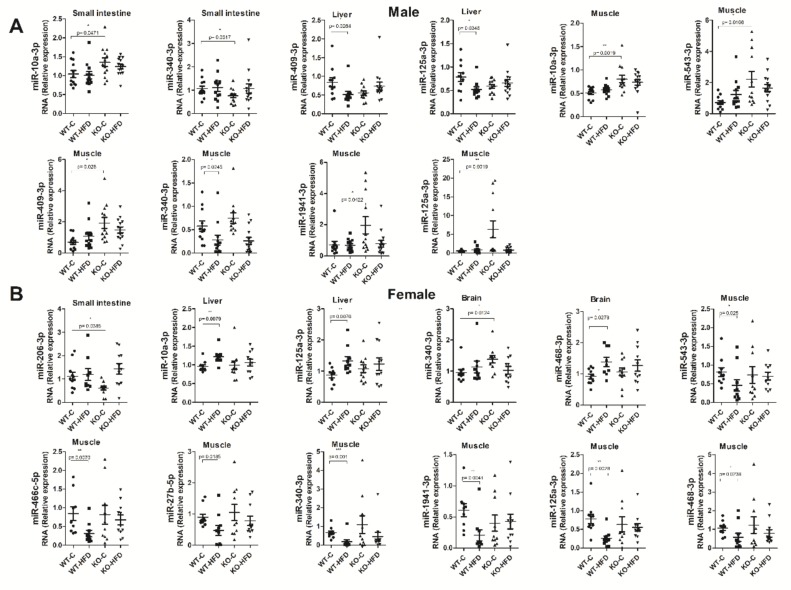
Tissue expression of miRNAs in response to oral high-fat dietary challenge (HFD). miRNAs modulated in postprandial lipemia in wild type (C57BL/6) mice and Dicer1-deficient mice 2 h after the HFD. RT-qPCR analysis of selected miRNAs in small intestine, liver, brain, and skeletal muscle. (**A**) Shows results for males and (**B**) for females. Statistical significance is indicated by * (*p* < 0.05), ** (*p* < 0.01), or *** (*p* < 0.001). C, Control; WT, wild type; KO, Dicer1-deficient mice.

**Figure 5:**
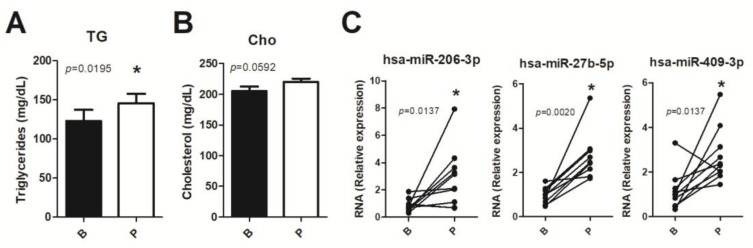
Postprandial circulating miRNAs in humans. Plasma levels of (**A**) triglycerides and (**B**) cholesterol under basal and postprandial conditions (*n* = 10 subjects). (**C**) Circulating miRNAs in human exosome samples. Values expressed as mean ± SEM. * Indicates a statistical difference at *p* < 0.05. TG, triglyceride; Cho, cholesterol; B, basal; P, postprandial.

Results are expressed as means ± SEM. The statistical significance (*p* < 0.05) of the miRNAs modulations was assessed by one-way analysis of variance (ANOVA) following all pairwise comparisons by Tukey–Kramer’s post hoc test (Table 1). Statistical analysis was performed using GraphPad Prism V.5 (San Diego, CA, USA) or GenEx Pro analysis software (MultiD Analyses AB, Sweden) for Figure 2. Time-course expression of circulating miRNAs in response to dietary fat challenge was assessed with a two-tailed unpaired *t*-test using the Wilcoxon test (Figure 3) to compare basal levels against each time point. Analysis of miRNA expression in tissues was performed with a two-tailed unpaired *t*-test using the Mann–Whitney test to compare each two groups (Figure 4). Plasma lipids and c-miRNAs in response to fat challenge (Figure 5) were assessed with a two-tailed paired *t*-test using the Wilcoxon matched test. 

## 3. Results and Discussion

### 3.1. miRNA Screening in Response to Dietary Fat Challenge

As postprandial lipemia is a dynamic, non-steady-state condition in which humans spend the majority of their time [34], miRNAs are potential candidates for therapeutic interventions [35]. We first searched c-miRNAs responding to an oral dietary fat challenge. Data from the initial screening, analyzing a list of unique 641 mice miRNAs, showed that only 435 miRNAs were detected in plasma samples of both genotypes two hours after the fat dietary challenge (Figure 2). Because of the relevance of intestinal miRNAs in systemic lipid metabolism in response to fat consumption [36], Dicer1 mutant mice were used in order to search for potential miRNAs secreted by this tissue. Seventy-three miRNAs showed differential expression between any of the treatments after the dietary challenge. These results prove our hypothesis that c-miRNAs could be modulated by postprandial lipemia (Figure 2A). Although not evaluated in their circulating form, a recent study carried out with peripheral blood mononuclear cells showed that diets enriched in saturated fatty acids modify miRNA expression [23], suggesting that postprandial lipemia also influences miRNA expression in tissues. In contrast to previous studies, where specific c-miRNAs were evaluated in a postprandial state [24,25], our data evaluate the whole mice miRNome for the first time. Figure 2B–D shows the differential miRNA expression between different genotypes in mice. The use of Dicer1 mutant mice led to the identification of potential miRNA candidates that might be secreted or influenced by the loss of Dicer1 in the intestine.

### 3.2. Validation of Modulated miRNAs 

Based on the data obtained in the screening, 68 c-miRNAs candidates with biological variability in mice (including WT and Dicer1 mutants; control and oral high-fat dietary challenge) were selected using one-way ANOVA for validation in a second animal cohort (Table 1). Supplementary Table S1 shows miRNA interactions according to *t*-test statistical significance. Only nine c-miRNAs showed statistically different expression in WT-HFD vs. WT-C (miR-1198-3p, miR-543-3p, miR-496a-3p, miR-466b-5p, miR-466c-5p, miR-206-3p, miR-1941-3p, miR-27b-5p, and miR-10a-3p). The association between miR-206-3p and target genes involved in the metabolism of lipids and glucose has been recently reported [37]. In addition, miR-206-3p and its target genes could be biomarker candidates to regulate fat excess [38]. This miRNA has been found to be upregulated in rat iliac artery under diabetic atherosclerosis [37]. Also, the upregulation of this miRNA can inhibit 3T3-L1 preadipocyte differentiation via the c-Met/PI3K/Akt signaling pathways [39]. Interestingly, miR-206-3p is a tissue-specific miRNA from skeletal muscle [40], and its overexpression in plasma has been associated with muscle slow-fiber damage in rats [41]. The involvement of this c-miRNA in atherosclerosis, which is induced after postprandial lipemia as well as in muscle damage, is intriguing and deserves further investigation. In addition, miR-466b-5p is upregulated under liver disease conditions [42] and is also overexpressed in infection and lipopolysaccharide-induced inflammation [43]. For the other validated miRNAs, there is scarce information with relation to lipid metabolism.

C-miRNAs might originate from different types of tissues, depending on different conditions. Knowing the specific tissue origin of miRNAs might contribute to the clarification of their functions and their roles and suggest them as potential targets for therapeutic modulation of miRNA secretion and disease progression control. Our study also aimed at searching potential circulating miRNAs released from the small intestine, and for that reason, we used Dicer1-deficient mice. Comparing the miRNA levels under basal condition between both genotypes, only six miRNAs were significantly validated (miR-542-3p, miR-10b-3p, miR-1198-3p, miR-10a-3p, miR-543-3p, and miR-329-3p), suggesting their possible intestinal influence (Supplementary Information Table S1). Borderline, nonsignificant modulation was found for miR-215-5p and miR-27b-5p. As previously described, Dicer1-deficient mice have a disorganized intestinal epithelium and present other malignancies such as increased in intestinal inflammation and impaired intestinal barrier function [44]. Proteomic studies elucidated the role of Dicer1 in intestinal lipid metabolism [45]. Whether the intestine directly contributes to their secretion or whether this condition is a consequence of Dicer1-deficient mice phenotype is unknown. Regarding the significantly modulated miRNAs, targets of miR-329-3p have been found to be related to lipid and glucose metabolism [37]. The other miRNAs are either not related to lipid metabolism or are involved in other metabolic pathways.

### 3.3. Kinetic of Validated miRNAs 

Based on the postprandial c-miRNAs validated in the second cohort of animals (Supplementary Table S1), the top 18 miRNAs were selected to study their kinetic response at *t* = 0, 0.5, 1, 2, and 4 h after HFD (Figure 3). miR-466b-5p was removed from the analysis because of inconsistent expression response. The studied c-miRNAs responded to oral fat challenge with different kinetics. While some responded immediately in the first 30 min, others were significantly modulated at later time points. 

From the 18 postprandial c-miRNAs, miR-215-5p and miR-496a-3p were not further analyzed due to statistical non-significance in the time-course analysis. However, an increasing trend was observed one hour after treatment. It is important to note that the magnitude of changes in the expression of c-miRNAs varies across the different animal cohorts, as observed here (Figure 3). Some miRNAs unique to mice were also identified, and thus probably lack biological relevance for humans (miR-466c-5p, miR-468-3p, miR-1198-3p, miR-1982-5p, and miR-1941-3p). From these five miRNAs, only three (miR-466c-5p, miR-468-3p, and miR-1941-3p) were further analyzed. From the other 11 miRNAs, only a limited number were selected for further analysis. In summary, a total of 10 miRNAs were selected (miR-206-3p, miR-10a-3p, miR-543-3p, miR-466c-5p, miR-27b-5p, miR-409-3p, miR-340-3p, miR-1941-3p, miR-125a-3p, and miR-468-3p).

### 3.4. Tissue Expression of miRNAs 

High-fat diets both negatively and positively impact miRNA expression (independent of body weight status). Therefore, the biological function of tissues such as adipose, skeletal, cardiac muscle, liver, neuronal, and endothelial tissue might be modulated by miRNAs [46]. For instance, in the small intestine, miRNAs are closely involved in energy homeostasis, lipid metabolism, and weight gain induced by high-fat diet. Diets rich in fats, apart from their detrimental effects on CVD, are associated with disturbances of the gastrointestinal transit, although these actions are not entirely clear [47]. To this end, we validated several miRNAs that were modulated by postprandial lipemia, although the source or fate of these miRNAs is not known. To gain insight into the possible sources or fates of these miRNAs, we selected a list of the 10 most consistently modulated miRNAs and evaluated their expression in different tissues (small intestine, liver, brain, and skeletal muscle) in the four experimental groups (Figure 4). We used tissues from cohort 1 and 2 (see Figure 1), which includes male and female mice sacrificed 2 h after the dietary fat challenge.

miRNAs levels in tissues were sex-dependent (Figure 4A,B). For instance, in the small intestine, the basal expression between genotypes of miR-10a-3p and miR-340-3p was statistically different between Dicer1-deficient mice vs. WT in males and miR-206-3p in females. The fat challenge did not affect the expression of the 10 analyzed miRNAs in the small intestine. 

Regarding the results obtained in the liver, miR-409-3p and miR-125a-3p expression was reduced as a consequence of the dietary challenge in male mice, while miR-10a-3p and 125a-3p were induced in females. Mice genotype did not affect the expression of these miRNAs in liver. 

Regarding brain, miR-468-3p expression was modulated according to the diet, i.e., increasing in females, while expression was modulated according to the genotype in the case of miR-340-3p. Interestingly, the expression of miR-468-3p in plasma was repressed two hours after the HFD, while its levels in the brain increased. Whether this reduction in plasma is compensated by brain uptake cannot be ascertained. Previous studies carried out on circulating brain-enriched miRNAs suggest their relevance in aging or neurodegenerative processes [48]. In addition, there are studies aimed at identifying miRNAs as biomarkers of brain disease associated with bad eating habits (e.g., cerebral ischemia) [49].

Finally, as the skeletal muscle participates in thermogenesis, lipid uptake, and other metabolic processes [50], the expression of the 10 final candidates was evaluated in this tissue. Skeletal muscle was the tissue presenting the greatest changes in expression in all the miRNAs analyzed. Interestingly, miR-543-3p, miR-340-3p, miR-466c-5p, miR-27b-5p, miR-1941-3p, miR-125a-3p, and miR-468-3p were downregulated after high-fat intake, especially in female mice. In male mice, miR-340-3p was also downregulated after the HFD. According to the genotype, miR-10a-3p, miR-543-3p, miR-409-3p, miR-1941-3p, and miR-125a-3p were upregulated in male mice. Detailed data on all analyzed miRNAs for all tissue are reported in Supplementary Figure S1 and Supplementary Table S2.

The miRNAs validated here have been previously associated with different types of diseases such as gastric cancer (miR-10a-3p, miR-27b-5p, and miR-409-3p) [51,52]. Nevertheless, caution is advised as the confirmation of any miRNA as a biomarker of any disease or specific tissue requires consistent results in numerous studies. 

### 3.5. Human miRNA Expression

In an attempt to validate the abovementioned miRNAs in human samples, we performed a pilot study with 10 young human healthy volunteers and evaluated c-miRNAs in response to a postprandial lipemia. As expected, three hours after the nutritional dietary challenge, TGs (Figure 5A) plasma levels were significantly increased, but not cholesterol (*p* = 0.0592) (Figure 5B). C-miRNAs can be transported in different molecules, including high-density lipoproteins (HDL), associated with Ago2 proteins and enclosed in extracellular vesicles such as exosomes [53]. Because of the relevance of exosomes as transporters of miRNAs and their role as disease biomarkers [54], we next analyzed, in exosomes, the expression of the validated candidates conserved in humans (miR 206-3p, miR-10a-3p, miR 543-3p, miR 409-3p, miR 27b-5p, miR 340-3p, and miR-125a-3p). Four miRNA candidates were not detected in human exosome samples. The three detected miRNAs (hsa-miR 206, hsa-miR 409-3p, and hsa-miR 27b-5p) were found to be upregulated in exosomes in postprandial lipemia. Our results show, in a postprandial setting, a change in the miRNA expression of miRNAs transported by extracellular vesicles, confirming that some miRNAs altered in mice were also modulated in humans.

### 3.6. Target Genes and Predictive Functionality of Postprandially Modulated miRNAs

**Figure 6:**
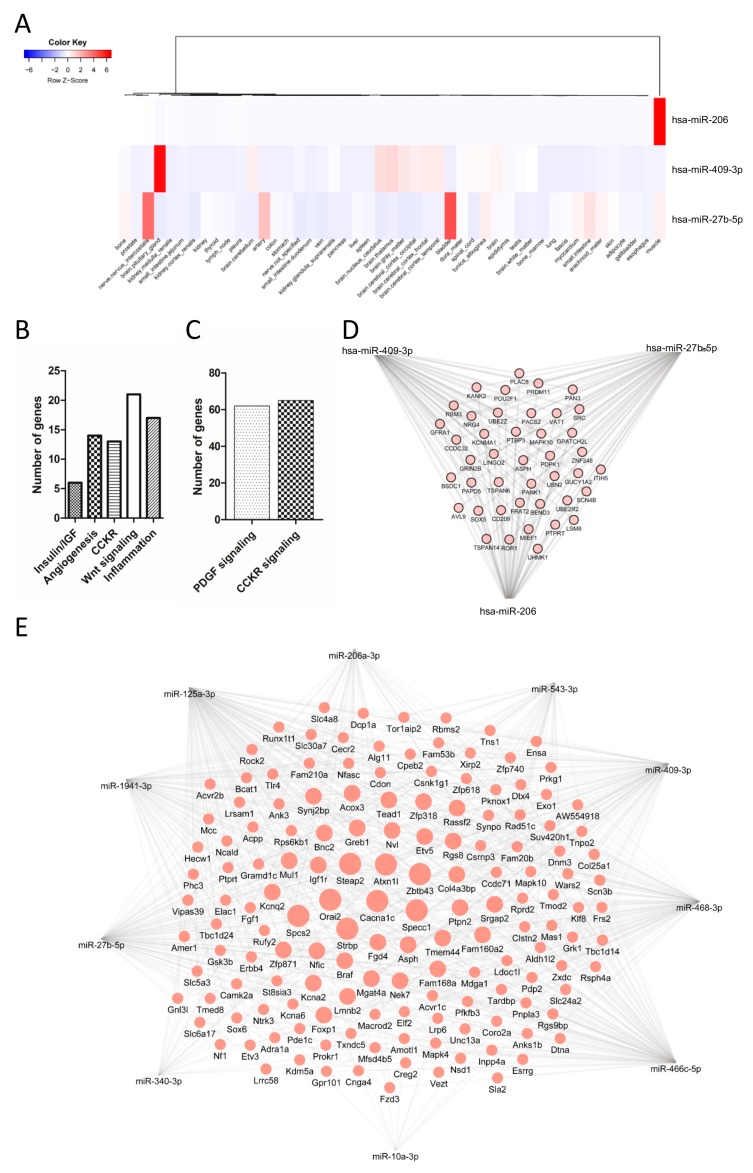
In silico functional analysis of postprandial circulating miRNAs. (**A**) In silico analysis of the possible tissue of origin of the c-miRNAs significantly modulated by the dietary fat challenge in humans and hierarchical clustering analysis. Panther pathways of over-represented pathways for (**B**) human and (**C**) mice target genes of modulated c-miRNAs. Gene interaction analysis for possible validated target genes potentially modulated by more than one c-miRNA that responded to dietary fat challenge in humans (**D**) and mice (**E**). Potential targets of miRNAs were obtained from public databases (see Section 2.6 for details). IGF, insulin like growth factor; CCKR, cholecystokinin B receptor signaling pathway; PDGF, platelet derived growth factor.

In silico analysis of possible tissue of origin and hierarchical clustering analysis of validated miRNAs was performed using the Tissue Atlas Database [32] (Figure 6A). miR-206 is a muscle-specific miRNA and its increase in postprandial lipemia suggests it is released from this tissue in response to a fat challenge, although no significant changes were observed in its tissue of origin (Figure 4B). Several other studies have reported their secretion upon muscle damage [55]. miR-409-3p is a brain-enriched miRNA with very high expression in the pituitary gland, but it is also expressed in other brain regions. Interestingly, the hypothalamic–pituitary–adrenal axis plays a critical role in the control of food intake and the pathogenesis of obesity [56]. Indeed, food intake is influenced by a system of physiologic signals and behavioral controls consisting of positive and negative sensory feedback mechanisms, regulated by a complex neuroendocrine system. The possible role of miRNAs in food intake has been recently addressed [57]. However, the function of c-miRNAs involved in food intake is poorly described. We found that the level of miR-409 was reduced in liver of male mice after HFD (Figure 4B). This miRNA has been previously reported to be involved in cancer pathways, associated with the Jak–Stat pathway [58], or dysregulated in certain neurological disorders [59]. Finally, the Tissue Atlas Database indicates that miR-27b-5p is enriched in the bladder, certain nerves (intercostals), and arteries. This miRNA has a widespread function in several aspects of lipid metabolism and adipocyte biology [60] and has been associated with exosomes in obesity [61].

We next performed a pathway analysis of validated c-miRNAs both in mice and humans using validated targets supported by strong experimental evidence obtained from the miRWalk database [62]. Data obtained from the Panther pathway analysis suggested the involvement of targets in insulin/insulin like growth factor (insulin/IGF) signaling, angiogenesis, cholecystokinin B receptor signaling pathway (CCKR), Wnt signaling and inflammation for mouse-validated miRNAs (Figure 6B), and in platelet derived growth factor (PDGF) and CCKR signaling for human postprandial validated miRNAs (Figure 6C). This data suggests that the potential targets of postprandial circulating miRNAs are involved in different metabolic processes.

A relevant feature of miRNA function is that one gene can be regulated by different miRNAs. To search for target genes potentially modulated by more than one circulating miRNA (modulated in postprandial lipemia) validated here in mice and humans, we performed a gene interaction (GI) analysis using validated target genes from databases (see Section 2 Material and Methods) (Figure 6D–E). Only genes targeted by three (humans) or four (mice) miRNAs are depicted. For mice, a unique list of 152 genes predicted to be regulated by more than 4 miRNAs contained Cacna1c, Specc1, Zbtb43, Strbp, Steap2, Orai2, Spcs2, Atxn1l, Igf1r, Col4a3bp, Fam160a2, Ptpn2, and Srgap2 among the most potentially regulated genes (Figure 6D). For humans, targets predicted to be regulated by the three miRNAs included 42 unique genes, including: Pdrm11, Pank1, Mapk10, Src, Ror1, Sox5, Ubn2, Znf248, and Pou2f1, among others (Figure 6E). Whether the validated circulating miRNAs described here are responsible for all the biological processes associated to postprandial lipid state deserves further investigation.

The strengths of our study are the use of different mice cohorts to validate the postprandial response of candidates, as well as the analysis of tissue levels of the candidate miRNAs to determine the possible source/fate of the circulating miRNAs. Moreover, some of the determinations (i.e., tissue distribution of modulated circulating miRNAs) were performed in male and female mice, suggesting an influence of sex on their possible function. Indeed, we found sex-dependent differential enrichment for certain miRNAs and for certain tissues. Our data add evidence to the possible role of circulating miRNAs to the well-known evidence of sex influence on systemic lipid metabolism [63]. 

Some limitations of the present study should be acknowledged. First, four of the validated miRNAs that are conserved in humans (miR-450a-2-3p, miR-542-3p, miR-183-3p, miR-130b-5p) and others from mice (miR-1198-3p, miR-1982) were not further analyzed. Whether these miRNAs are relevant in tissues or postprandial lipemia is not known and would need further investigation. Second, the analysis of tissue levels of c-miRNAs can only provide an idea of their enrichment or depletion of the selected miRNAs. Third, the four c-miRNAs not detected in humans (miR-10a-3p, miR-543-3p, miR-340-3p, and miR-125a-3p) were shown to be absent only in exosomes. We cannot exclude the possibility that these miRNAs can be transported by other means not evaluated here (i.e., associated with lipoproteins, Ago2, or others) and might also respond to postprandial lipemia in humans. Moreover, in the human study, no samples were taken at other time points during the postprandial response. We do not rule out the possibility that postprandially induced changes in miRNA levels are not linear, as with other plasma biomarkers that respond to postprandial perturbations [64]. Fourth, the initial screening was performed in 20 animals (n = 5 per group), which might represent a source of potential bias over the most differentially modulated miRNAs. We do not discard the notion that more candidates could be presented if a larger number of animals were used in the screening phase. Fifth, 641 circulating miRNAs were screened by RT-qPCR, which represent the most abundant mice miRNAs. Other less common miRNAs described in the recently released miRBase V.22 were not screened. In this aspect, a screening performed by RNA sequencing could give more information of novel or less abundant miRNAs. Finally, we can only speculate about the potential role of c-miRNAs as mediators of the molecular response to postprandial lipemia. Mechanistic in vitro and in vivo studies are necessary to experimentally validate the function of each of the postprandial c-miRNA candidates.

## 4. Conclusions

In summary, our results add novel evidence concerning the specific c-miRNA profile induced by dietary fat challenges. Moreover, we also provide evidence that certain c-miRNAs are influenced by the loss of Dicer1 in the intestine, which points to the role of intestinal miRNAs in systemic lipid metabolism. Their precise origin, fate, and mechanisms of action (if any) deserve further investigation. In this sense, our data also show that the intestine, liver, brain, or skeletal muscle could be the source or target of certain postprandial c-miRNAs. Understanding the potential role of c-miRNAs as mediators of the molecular response to postprandial lipemia can help identify novel targets for CVD prevention or treatment through the therapeutic modulation of miRNA function. 

## Figures and Tables

**Table 1:** Validation of circulating miRNAs modulated by oral high-fat diet challenge and genotype.

miRNA	*p*-Value	miRNA	*p*-Value
miR-466b-5p	1.36 × 10^−5^	miR-208a-5p	0.403192
miR-206-3p	9.30 × 10^−5^	miR-137-3p	0.407196
miR-10a-3p	0.000143	miR-127-5p	0.415694
miR-1198-3p	0.000287	miR-762	0.44027
miR-543-3p	0.000857	miR-489-3p	0.443628
miR-450a-2-3p	0.000977	miR-470-3p	0.48737
miR-466c-5p	0.001046	miR-20a-3p	0.510021
miR-215-5p	0.003314	miR-700-3p	0,524019
miR-27b-5p	0.003793	miR-34b-5p	0.541163
miR-409-3p	0.006274	miR-291a-3p	0.565681
miR-1982-5p	0.01161	miR-153-3p	0.585034
miR-496a-3p	0.013289	miR-467c-5p	0.586
miR-340-3p	0.027317	miR-671-3p	0.596674
miR-183-3p	0.030129	miR-291a-5p	0.597004
miR-1941-3p	0.03114	miR-615-3p	0.602924
miR-130b-5p	0.041939	miR-376c-5p	0.619959
miR-125-3p	0.050039	miR-182-5p	0.623672
miR-1251	0.053334	miR-1b-5p	0.62594
miR-542-3p	0.063337	miR-299a-3p	0.629587
miR-10b-3p	0.147649	miR-490-5p	0.630007
miR-1943-5p	0.201154	miR-1927	0.630042
miR-329-3p	0.21081	miR-380-3p	0.63362
miR-20b-3p	0.211707	miR1894-3p	0.656185
miR-680	0.217664	miR-342-5p	0.668416
miR-331-5p	0.288161	miR-342-5p	0.678952
miR-1186a	0.29046	miR-325-3p	0.693872
miR-335-3p	0.294498	miR-21a-3p	0.704326
miR-804	0.31838	miR-743b-5p	0.724951
miR-667-3p	0.325072	miR-216a-5p	0.758007
miR-468-3p	0.371391	miR-323-3p	0.774188
miR-291b-5p	0.373164	miR-129-5p	0.791411
miR-295-5p	0.390927	miR-1953	0.834637
miR-194-2-3p	0.396934	miR-207	0.892798
miR-875-5p	0.401872	miR-6691-5p	0.92054

Notes: miRNA expression of WT-C, WT-HFD, KO-C, and KO-HFD, analyzed by RT-qPCR. One-way analysis of variance (ANOVA) analysis was used for multiple comparisons using GenEx Pro analysis software (MultiD Analyses AB, Sweden). Male and female mice were included in each group (*n* = 47 in total). WT, wild type; KO, Dicer1-deficient mice; C, control; HFD, oral dietary fat challenge. *p*-values are changes across any experimental groups for any of the multiple comparison test.

## Supplementary Materials

The following are available online at https://www.mdpi.com/2072-6643/11/6/1326/s1, Table S1: Fold change and statistical values between the different groups. Table S2: Validation of circulating miRNAs modulated by oral high fat diet challenge and genotype in target tissues. Figure S1: Tissue expression of miRNAs in response to oral high fat dietary challenge (HFD) and genotype.
