# Supplementary material for: Postprandial Circulating miRNAs in Response to a Dietary Fat Challenge
Source: Nutrients. 2019 Jun 13;11(6):1326. doi: 10.3390/nu11061326 (PMC6627817; doi:10.3390/nu11061326)
Supplement: Supplementary file 1 [file nutrients-11-01326-s001.pdf]

Postprandial circulating miRNAs in response to a dietary fat challenge

Diana C. Mantilla-Escalante[1§], María-Carmen López de las Hazas[1§], Judit Gil-Zamorano[1], Lorena del Pozo-Acebo[1], M. Carmen Crespo[2], Roberto Martín-Hernández[3], Andrea del Saz[1], Joao Tomé-Carneiro[2], Fernando Cardona[4,5], Isabel Cornejo-Pareja[4,5], Almudena García-Ruiz[1], Oliver Briand[6], Miguel A. Lasunción[7,5], Francesco Visioli[2,8], Alberto Dávalos[1*].

[1] Laboratory of Epigenetics of Lipid Metabolism, Madrid Institute for Advanced Studies (IMDEA)-Food, CEI UAM + CSIC, 28049 Madrid, Spain.

[2] Laboratory of Functional Foods, Madrid Institute for Advanced Studies (IMDEA)-Food, CEI UAM + CSIC, 28049 Madrid, Spain.

[3]GENYAL Platform on Nutrition and Health, Madrid Institute for Advanced Studies (IMDEA)-Food, CEI UAM + CSIC, 28049 Madrid, Spain.

[4]Unidad de Gestión Clínica de Endocrinología y Nutrición del Hospital Virgen de la Victoria, Instituto de Investigación Biomédica de Málaga (IBIMA), Universidad de Málaga, 29010 Málaga, Spain.

[5]CIBER Fisiopatología de la Obesidad y Nutrición (CIBEROBN), Instituto de Salud Carlos III, 28029 Madrid, Spain

[6]University Lille, Inserm, Centre Hospitalier Universitaire (CHU) de Lille, Institut Pasteur de Lille, U1011-European Genomic Institute for Diabetes, Lille, France

[7]Servicio de Bioquímica Investigación, Hospital Universitario Ramón y Cajal, IRYCIS, 28034 Madrid, Spain

[8]Department of Molecular Medicine, University of Padova, 35121 Padova, Italy

[§]These authors contribute equally to this work.
Correspondence to: Dr. Alberto Dávalos (E-mail: alberto.davalos@imdea.org)
Laboratory of Epigenetics of Lipid Metabolism, IMDEA Food Institute, CEI UAM + CSIC. Ctra. De Cantoblanco 8, 28049 Madrid. Spain. Phone +34912796985.

Supplementary materials

Table S1. Fold change and statistical values between the different groups.

(WT-HFD) vs (WT-C)	Fold change	Difference (A-B log scale)	*P*-Value
miR-1198-3p	-5,12556	-2,35771	0,001145
miR-543-3p	2,34323	1,2285	0,001904
miR-496a-3p	2,40848	1,26812	0,004077
miR-466b-5P	23,18967	4,53541	0,005546
miR-466c-5p	-2,94844	-1,55995	0,008714
miR-206-3p	3,24399	1,69777	0,02169
miR-1941-3p	1,9238	0,94396	0,025058
miR-10a-3p	1,95404	0,96646	0,039038
miR-27b-5p	2,17692	1,12229	0,055025

(KO-C vs (WT-C)	Fold change	Difference (A-B log scale)	*P*-Value
miR-542-3p	1,99363	0,9954	0,002762377
miR-10b-3p	-2,53319	-1,34095	0,007380218
miR-1198-3p	-3,56505	-1,83392	0,014711768
miR-10a-3p	2,22383	1,15305	0,018572132
miR-543-3p	2,06838	1,0485	0,030816547
miR-329-3p	1,77958	0,83153	0,038015344
miR-215-5p	-1,89279	-0,92051	0,054891192
miR-27b-5p	2,11795	1,08267	0,079897461
miR-340-3p	-1,88773	-0,91665	0,116419803
miR-1941-3p	1,78538	0,83623	0,122616052

(KO-HFD) vs (KO-C)	Fold change	Difference (A-B log scale)	*P*-Value
miR-340-3p x	3,59091	1,84435	0,000147601
miR-206-3p	4,56648	2,19108	0,000371169
miR-466b-5P	-36,4061	-5,18611	0,000459668
miR-183-3p x	1,68699	0,75445	0,003694897
miR-409-3p x	2,06042	1,04294	0,005111443
miR-1198-3p	3,49946	1,80713	0,007450815
miR-10a-3p	2,04361	1,03112	0,027009354
miR-215-5p x	1,61759	0,69384	0,037291226
miR-1982-5p x	1,71945	0,78195	0,043227022
miR-208a-5p x	1,63599	0,71016	0,047238356
miR-489-3p x	1,6488	0,72142	0,049226081
miR-27b-5p	1,84548	0,884	0,061475501

(KO-HFD) vs (WT-HFD)	Fold change	Difference (A-B log scale)	*P*-Value
miR-466b-5P	-197,677	-7,627	8,92E-07
miR-450a-2-3p	4,88844	2,28938	1,46E-05
miR-466c-5p	3,32568	1,73365	0,000188986
miR-1198-3p	5,03126	2,33092	0,000343919
miR-1982-5p	2,40501	1,26604	0,000748593

miR-130b-5p	-2,92701	-1,54943	0,00287608
miR-206-3p	2,49963	1,32172	0,004796305
miR-10a-3p	2,32577	1,21771	0,008769936
miR-215-5p	-1,85612	-0,89229	0,014892181
miR-1943-5p	2,29808	1,20043	0,019565644
miR-125-3p	1,66176	0,73271	0,02345629
miR-680	1,86337	0,89791	0,024677988
miR-183-3p	1,4433	0,52938	0,026224037
miR-340-3p	3,21687	1,68566	0,029615939
miR-804	1,76362	0,81854	0,03566283
miR-667-3p	-1,68641	-0,75396	0,048039196
miR-27b-5p	1,79549	0,84437	0,054959703

WT, Wild type; KO, *Dicer1*-deficient mice; C, control; HFD, oral high fat dietary fat challenge. n=47 mice. miRNA analysis by RT-qPCR, before and after two hours of dietary fat challenge.

Table S2: Validation of circulating miRNAs modulated by oral high fat diet chellenge and genotype in target tissues.

miRNAs	Males				Females			
	WT-C	WT-HFD	KO-C	KO-HFD	WT-C	WT-HFD	KO-C	KO-HFD
	small intestine							
miR-206	1,11± 0,46	1,56 ± 0,58	1,85 ± 1,09	1,55 ± 0,76	1,12 ± 0,54	1,19 ± 0,72	0,62 ± 0,27	1,43 ± 0,72
miR-10a-3p	1,04 ± 0,31	1,01 ± 0,32	1,35 ± 0,40	1,24 ± 0,22	1,04 ± 0,29	1,20 ± 0,44	1,25 ± 0,91	1,32 ± 0,36
miR-543-3p	1,09 ± 0,45	1,34 ± 0,63	1,15 ± 0,79	1,26 ± 0,46	1,14 ± 0,58	1,10 ± 0,49	0,76 ± 0,53	1,11 ± 0,57
miR-466-5p	0,20 ± 0,13	0,26 ± 0,10	0,22 ± 0,10	0,24 ± 0,15	1,07 ± 0,44	1,39 ± 0,85	0,76 ± 0,39	1,68 ± 1,04
miR-27b-5p	1,04 ± 0,28	1,16 ± 0,31	1,12 ± 0,29	1,03 ± 0,36	1,07 ± 0,38	1,12 ± 0,28	1,15 ± 0,59	1,13 ± 0,36
miR-409-3p	1,07 ± 0,40	1,02 ± 0,38	1,49 ± 0,57	1,71 ± 0,78	1,11 ± 0,56	0,95 ± 0,26	0,88 ± 0,40	1,23 ± 0,44
miR-340-3p	1,07 ± 0,37	1,11 ± 0,55	0,77 ± 0,30	1,07 ± 0,70	1,07 ± 0,41	1,13 ± 0,44	0,88 ± 0,61	0,99 ± 0,53
miR-1941-3p	1,09 ± 0,39	1,44 ± 0,68	1,65 ± 0,70	1,75 ± 0,89	1,14 ± 0,60	1,02 ± 0,22	0,95 ± 0,48	1,21 ± 0,61
miR-125a-3p	1,14 ± 0,58	2,16 ± 2,05	2,22 ± 2,02	1,20 ± 0,45	1,20 ± 0,74	2,38 ± 2,91	0,81 ± 0,34	2,31 ± 1,74
miR-468-3P	1,06 ± 0,33	1,12 ± 0,37	1,01 ± 0,49	1,22 ± 0,52	1,11 ± 0,48	1,05 ± 0,31	1,05 ± 0,47	1,19 ± 0,57
Liver								
miR-206	0,83 ± 0,57	0,65 ± 0,31	1,05 ± 0,65	0,87 ± 0,63	0,94 ± 0,60	1,40 ± 0,75	0,87 ± 0,56	1,56 ± 1,38
miR-10a-3p	0,92 ± 0,23	0,77 ± 0,24	0,75 ± 0,13	0,86 ± 0,26	0,96 ± 0,14	1,22 ± 0,18	0,99 ± 0,36	1,06 ± 0,29
miR-543-3p	0,85 ± 0,58	0,63 ± 0,17	1,08 ± 0,60	0,80 ± 0,46	0,89 ± 0,53	1,21 ± 0,58	1,02 ± 0,62	1,05 ± 0,74
miR-466-5p	0,99 ± 0,86	0,58 ± 0,20	1,08 ± 0,76	0,74 ± 0,57	1,01 ± 0,76	1,36 ± 1,03	1,02 ± 0,81	1,32 ± 1,39
miR-27b-5p	0,90 0,77	0,74 ± 0,33	1,07 ± 0,77	0,66 ± 0,64	0,93 ± 0,52	1,14 ± 0,77	0,72 ± 0,39	0,84 ±0,60
miR-409-3p	0,84 ± 0,38	0,52 ± 0,27	0,56 ± 0,18	0,74 ± 0,38	0,88 ± 0,28	1,13 ± 0,24	1,12 ± 0,65	1,07 ± 0,36
miR-340-3p	0,74 ± 0,23	0,72 ± 0,14	0,88 ± 0,29	0,70 ± 0,17	0,88 ± 0,15	1,04 ± 0,32	0,98 ± 0,30	1,05 ± 0,40
miR-1941-3p	0,85 ± 0,57	0,77 ± 0,59	0,97 ± 0,68	0,75 ± 0,49	0,87 ± 0,50	1,28 ± 0,85	1,05 ± 0,65	1,19 ± 1,05
miR-125a-3p	0,79 ± 0,30	0,52 ± 0,18	0,59 ± 0,14	0,66 ± 0,26	0,88 ± 0,25	1,33 ± 0,41	1,07 ± 0,39	1,22 ± 0,65
miR-468-3P	0,82 ± 0,47	0,69 ± 0,24	0,93 ± 0,39	0,84 ± 0,41	0,91 ± 0,53	1,22 ± 0,65	0,94 ± 0,51	1,07 ± 0,81
Brain								
miR-206	1,07 ± 0,44	1,06 ± 0,46	1,59 ± 1,13	1,41 ± 0,77	1,02 ± 0,50	1,53 ± 1,01	1,11 ± 0,51	1,26 ± 0,65
miR-10a-3p	1,15 ± 0,56	0,89 ± 0,40	1,38 ± 0,98	1,26 ± 0,69	1,12 ± 0,68	1,02 ± 0,76	1,62 ± 1,46	0,95 ± 0,72
miR-543-3p	1,08 ± 0,40	1,19 ± 0,54	1,40 ± 0,76	1,54 ± 0,62	1,01 ± 0,33	1,30 ± 0,77	1,15 ± 0,59	1,05 ± 0,55
miR-466-5p	1,18 ± 0,71	1,52 ± 1,25	1,95 ± 1,97	1,83 ± 1,03	0,95 ± 0,44	1,58 ± 0,87	1,13 ± 0,62	1,39 ± 0,86
miR-27b-5p	1,05 ± 0,32	1,13 ± 0,42	1,49 ± 0,86	1,39 ± 0,51	1,02 ± 0,45	1,27 ± 0,55	1,27 ± 0,56	1,22 ± 0,69
miR-409-3p	1,04 ± 0,29	1,15 ± 0,72	1,35 ± 1,05	1,40 ± 0,73	0,96 ± 0,38	1,33 ± 0,70	1,23 ± 0,55	1,16 ± 0,45
miR-340-3p	1,80 ± 0,39	2,33 ± 1,53	2,61 ± 1,19	2,68 ± 1,21	0,95 ± 0,29	1,13 ± 0,53	1,38 ± 0,37	1,03 ± 0,39
miR-1941-3p	0,27 ± 0,18	0,34 ± 0,19	0,46 ± 0,47	0,44 ± 0,30	1,04 ± 0,57	1,78 ± 1,26	1,16 ± 0,64	1,42 ± 0,84
miR-125a-3p	1,12 ± 0,53	1,06 ± 0,46	1,37 ± 0,74	1,37 ± 0,83	1,12 ± 0,54	1,28 ± 0,72	1,16 ± 0,84	1,33 ± 0,70
miR-468-3P	1,08 ± 0,44	1,27 ± 0,90	1,39 ± 1,10	1,65 ± 0,76	0,92 ± 0,23	1,39 ± 0,43	1,07 ± 0,39	1,27 ± 0,58
Muscle								
miR-206	0,78 ± 0,36	1,14 ± 0,85	1,07 ± 0,78	1,09 ± 0,42	1,01 ± 0,42	0,69 ± 0,68	1,13 ± 0,79	1,10 ± 0,66
miR-10a-3p	0,51 ± 0,12	0,57 ± 0,11	0,81 ± 0,28	0,74 ± 0,23	0,64 ± 0,33	0,74 ± 0,26	0,96 ± 0,80	0,63 ± 0,17

miR-543-3p	0,72 ± 0,42	1,23 ± 0,88	2,21 ± 1,69	1,65 ± 0,84	0,81 ± 0,36	0,45 ± 0,47	0,73 ± 0,66	0,70 ± 0,33
miR-466-5p	0,89 ± 0,98	2,04 ± 2,43	4,53 ± 5,64	1,13 ± 0,86	0,84 ± 0,53	0,31 ± 0,26	0,81 ± 0,76	0,68 ± 0,42
miR-27b-5p	0,73 ± 0,29	1,01 ± 0,78	1,04 ± 0,64	0,91 ± 0,36	0,89 ± 0,31	0,47 ± 0,49	1,05 ± 0,78	0,78 ± 0,48
miR-409-3p	0,69 ± 0,44	1,07 ± 0,82	1,92 ± 1,18	1,47 ± 0,71	0,72 ± 0,29	0,51 ± 0,62	0,94 ± 1,10	0,73 ± 0,52
miR-340-3p	0,58 ± 0,35	0,28 ± 0,35	0,75 ± 0,36	0,26 ± 0,27	0,67 ± 0,28	0,19 ± 0,32	1,10 ± 1,41	0,46 ± 0,74
miR-1941-3p	0,71 ± 0,72	0,68 ± 0,37	1,96 ± 1,81	0,78 ± 0,78	0,61 ± 0,30	0,20 ± 0,26	0,40 ± 0,39	0,4 ± 0,37
miR-125a-3p	0,58 ± 0,26	0,84 ± 0,83	6,33 ± 7,41	0,73 ± 0,67	0,78 ± 0,38	0,26 ± 0,20	0,63 ± 0,61	0,55 ± 0,32
miR-468-3P	0,97 ± 0,65	1,35 ± 1,13	1,66 ± 0,88	1,62 ± 0,80	1,07 ± 0,36	0,57 ± 0,66	1,23 ± 1,35	0,79 ± 0,63

Tissue expression of miRNAs in response to oral high fat dietary challenge (HFD). miRNAs modulated in postprandial lipemia in Wild type (C57BL/6) mice and Dicer1- deficient mice 2 hours after the HFD. RT-qPCR analysis of selected miRNAs in small intestine, liver, brain, and skeletal muscle. The results represent the average of the ΔΔct.

Figure S1: Tissue expression of miRNAs in response to oral high fat dietary change and genotype.

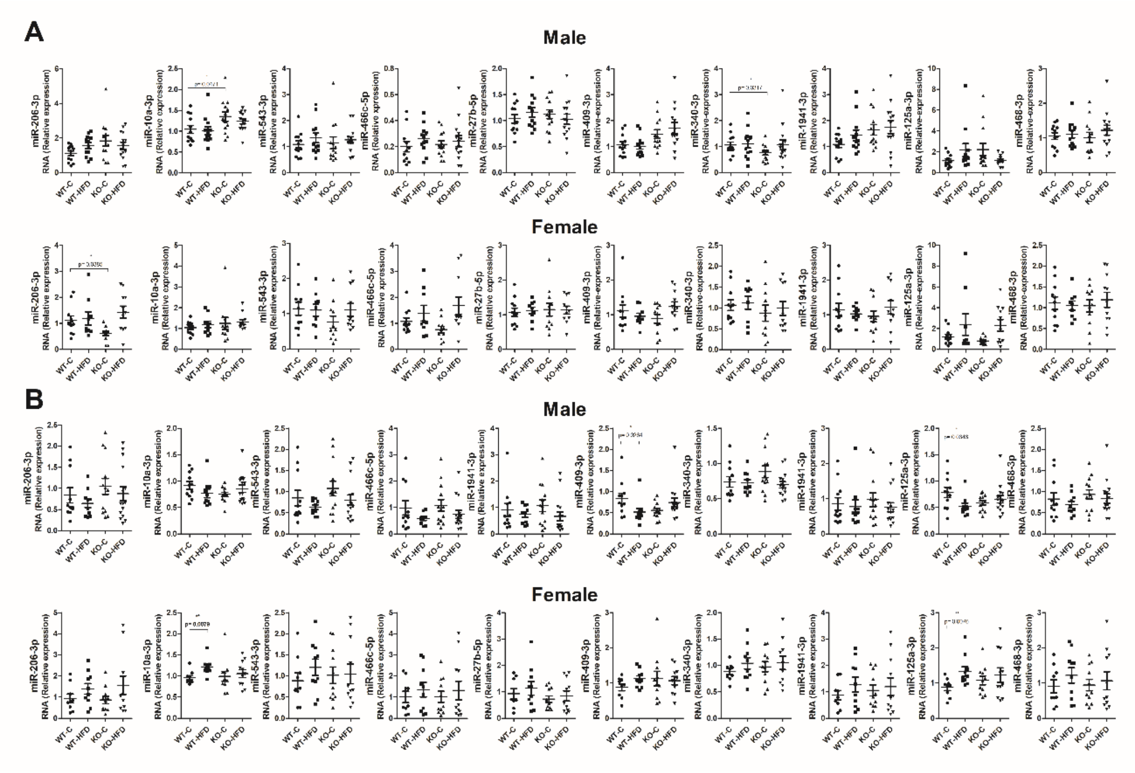

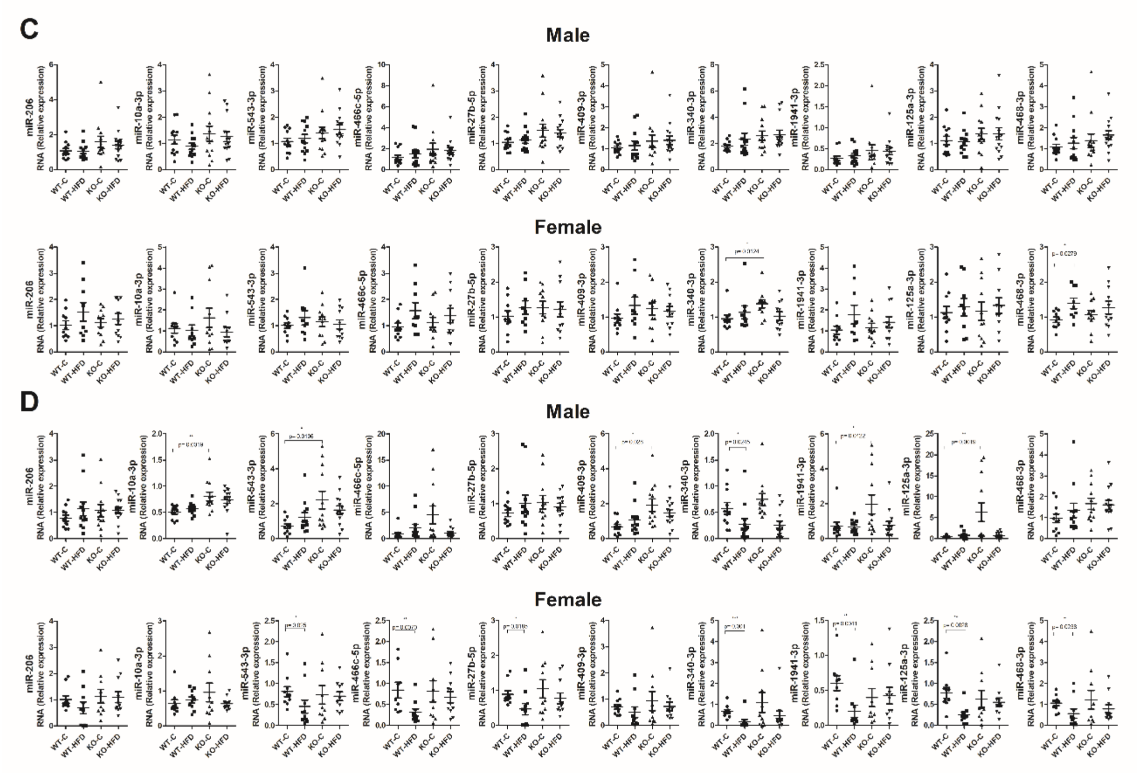

Tissue expression of miRNAs in response to oral high fat dietary challenge (HFD). miRNAs modulated in postprandial lipemia in Wild type (C57BL/6) mice and Dicer1- deficient mice 2 hours after the HFD. RT-qPCR analysis of selected miRNAs in (A) small intestine, (B) liver, (C) brain, and (D) skeletal muscle. *Indicates statistical significance at $p < 0.05$. C, Control; WT, Wild type; KO, Dicer1-deficient mice.
